# Morphine Does Not Affect Myocardial Salvage in ST-Segment Elevation Myocardial Infarction

**DOI:** 10.1371/journal.pone.0170115

**Published:** 2017-01-12

**Authors:** Hye Bin Gwag, Taek Kyu Park, Young Bin Song, Eun Kyoung Kim, Woo Jin Jang, Jeong Hoon Yang, Joo-Yong Hahn, Seung-Hyuk Choi, Jin-Ho Choi, Sang Hoon Lee, Yeon Hyeon Choe, Joonghyun Ahn, Keumhee Chough Carriere, Hyeon-Cheol Gwon

**Affiliations:** 1 Division of Cardiology, Department of Internal Medicine, Heart Vascular Stroke Institute, Samsung Medical Center, Sungkyunkwan University School of Medicine, Seoul, Republic of Korea; 2 Division of Cardiology, Department of Internal Medicine, Samsung Changwon Hospital, Sungkyunkwan University School of Medicine, Changwon, Republic of Korea; 3 Department of Radiology, Samsung Medical Center, Sungkyunkwan University School of Medicine, Seoul, Republic of Korea; 4 Department of Biostatistics and Clinical Epidemiology, Samsung Medical Center, Seoul, Republic of Korea; 5 Department of Mathematical and Statistical Sciences, University of Alberta, Edmonton, Alberta, Canada; Azienda Ospedaliero Universitaria Careggi, ITALY

## Abstract

Recent studies have proposed intravenous (IV) morphine is associated with delayed action of antiplatelet agents in acute myocardial infarction. However, it is unknown whether morphine results in increased myocardial damage in ST-segment elevation myocardial infarction (STEMI) patients undergoing primary percutaneous coronary intervention (PCI). We investigated myocardial salvage index (MSI) to determine whether IV morphine affects myocardial injury adversely in STEMI patients undergoing primary PCI. 299 STEMI patients underwent contrast-enhanced magnetic resonance imaging a median of 3 days after PCI. Infarct size was measured on delayed-enhancement imaging, and area at risk was quantified on T2-weighted imaging. MSI was calculated as ‘[area at risk–infarct size] X 100 / area at risk’. IV morphine was administrated in 32.1% of patients. Patients treated with morphine had shorter symptom to balloon time and higher prevalence of Thrombolysis in Myocardial Infarction flow grade 0 or 1. The morphine group showed a trend toward larger MSI and infarct size and significantly greater area at risk than the non-morphine group. After propensity score matching (90 pairs), MSI was similar between the morphine and non-morphine group (46.1% versus 43.5%, *P* = .11), and infarct size and area at risk showed no difference. In propensity score-matched analysis, IV morphine prior to primary PCI in STEMI patients did not cause adverse impacts on myocardial salvage.

## Introduction

Intravenous (IV) morphine is recommended in patients with ST-segment elevation myocardial infarction (STEMI) when chest pain is unresponsive to nitrates [1,2]. However, this recommendation is not based on any prospective randomized clinical trials, rather only on expert opinion [1,2]. Furthermore, several recent studies have shown that IV morphine administration can decrease the effect of P2Y_12_ receptor inhibitors on platelet aggregation in healthy volunteers [3,4] and patients with acute myocardial infarction by causing delayed absorption in the gastrointestinal tract [5–7]. Considering the importance of adequate and rapid platelet inhibition in STEMI patients undergoing primary percutaneous coronary intervention (PCI), drug-drug interactions between P2Y_12_ receptor inhibitors and morphine may increase the risk of thrombotic events and adversely impact myocardial injury and salvage in the thrombogenic milieu of STEMI patients. Cardiac magnetic resonance (CMR) can provide precise pathologic information on infarct-related myocardial edema, infarcted myocardium, microvascular obstruction (MVO), and myocardial hemorrhage in the setting of STEMI [8]. In addition, CMR can quantify the extent of salvaged myocardium, and thus could provide a better understanding of the effects of IV morphine on myocardial injury in STEMI patients. Therefore, we investigated the effect of IV morphine on myocardial salvage assessed by CMR in STEMI patients undergoing primary PCI.

## Methods

**Fig 1:**
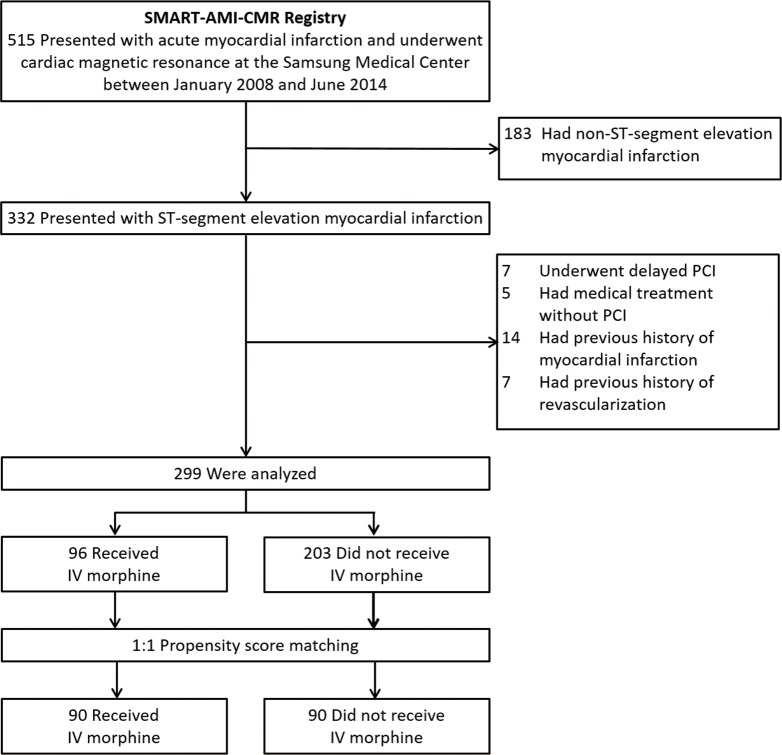
Study population.

The study population was selected from the Samsung Medical Center SMART-AMI-CMR registry. Between January 2008 and June 2014, 515 consecutive patients who presented with acute myocardial infarction and underwent CMR were enrolled in this registry. Among these patients, STEMI patients (n = 332), whose electrocardiogram showed ST-segment elevation more than 1 mm in two or more contiguous leads or a presumably new-onset left bundle branch block, were included in the present study. We excluded patients with symptom-to-balloon time more than 12 hours (n = 7), medical treatment without primary PCI (n = 5), or previous history of myocardial infarction (n = 14) or revascularization (n = 7). The final sample size for the study was 299 patients (Fig 1). The Institutional Review Board of Samsung Medical Center approved this study, and all subjects provided written informed consent to participate. Decisions regarding whether to administer IV morphine prior to PCI or not were made by the respective operators.

Before the PCI, all patients received 300 mg of aspirin and 600 mg of clopidogrel as loading doses if they had not previously taken those medications. Procedures including thrombus aspiration, predilation before stenting, use of glycoprotein IIb/IIIa inhibitors, and selection of size and the type of angioplasty balloon or stent were left to the operators’ discretion.

We used a 1.5 T scanner (Magnetom Avanto, Syngo MR B15 version; Siemens Medical Solutions, Erlangen, Germany) with a 32-channel phased array receiver coil. Cine images were acquired using a steady-state free-precession sequence with 8–10 contiguous short-axis slices to cover the entire left ventricle (LV) with a slice thickness of 6 mm and a 4 mm gap. T2-weighted image was performed in the cardiac short-axis direction using a black-blood T2-weighted inversion recovery fast spin echo sequence and delayed gadolinium-enhanced imaging was acquired with the phase sensitive inversion recovery technique after injection of 0.15 mmol/kg Gadovist (gadobutrol; Bayer Schering Pharma, Berlin, Germany) in 10–12 continuous short-axis images of 6 mm in thickness with a 4-mminter-slice gap. Delayed hyperenhancement and extent of microvascular obstruction (MVO) were evaluated 10 min after gadolinium administration by using a multi-shot turbo field echo breath-hold sequence [9].

**Fig 2:**
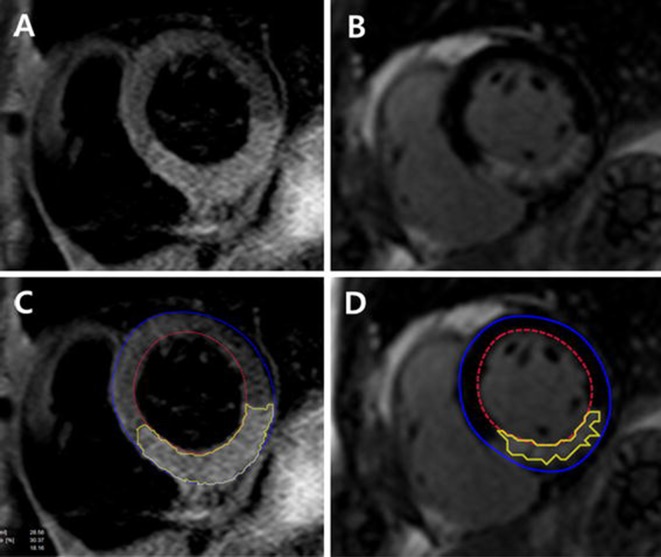
Example CMR images of reperfused inferior STEMI. Short-axis slices of T2-weighted image (A) and the corresponding delayed hyperenhancement image (B) in patients with inferior ST-segment elevation myocardial infarction. The extent of area at risk (C) and infarct size (D) are indicated as by yellow lines.

All measurements were performed at our CMR core laboratory. LV volume analysis was performed using commercialized software (CAAS MRV version 1.0, Pie Medical Imaging B.V., The Netherlands). The endocardial and epicardial borders were manually traced and papillary muscles and LV trabeculae were excluded from the endocardium [10]. LV mass was calculated by multiplying the myocardial volume by the myocardial density (1.05 g/mL). The T2-weighted image was used to determine the presence of hemorrhage [11] and quantification of area at risk (AAR). Hyperenhanced area was specified as infarct area and hypoenhanced region surrounded by the hyperenhanced area was considered as a sign of MVO. The volume of delayed hyperenhancement was calculated as the summation of the area of delayed hyperenhancement within each segment multiplied by 10 mm [12]. The proportion of delayed hyperenhancement to LV myocardial volume was defined as infarct size. The extent of MVO was calculated in the same manner. The myocardial salvage index (MSI) was calculated as (AAR—infarct size) x 100/AAR [13]. Fig 2 shows an example case.

Baseline characteristics, angiographic and procedural findings, CMR, and clinical outcome data were recorded prospectively by research coordinators as part of a dedicated registry [14]. Killip classification was determined before primary PCI. The creatine kinase-myocardial band fraction (CK-MB) was measured before PCI, and every 8 hours after the index procedure until a peak value was confirmed. Then CK-MB was measured once daily until the level was normalized. LV ejection fraction was measured by transthoracic echocardiography using Simpson’s methods before or immediately after primary PCI. Myocardial blush grade (MBG) was evaluated using the angiogram obtained at the end of the index procedures, as previously described: 0 = absence of contrast opacification in the myocardial infarct zone or persistent staining without washout; 1 = minimal contrast opacification; 2 = reduced but clearly evident blush in the infarct zone compared to the ipsilateral or contralateral noninvolved epicardial vessels; and 3 = myocardial contrast filling equal to or greater than that seen in the noninvolved epicardial vessels [15]. Major adverse cardiac events (MACEs) were defined as a composite of cardiac death, recurrent MI, ischemic stroke, and coronary revascularization after index procedure.

The primary outcome was MSI assessed by CMR. Secondary outcomes included AAR, myocardial infarct size, MVO, and presence of hemorrhagic infarction assessed by CMR and MACEs. Post-procedural Thrombolysis in Myocardial Infarction (TIMI) flow grade, post-procedural MBG, and enzymatic infarct size were also compared between morphine and non-morphine groups.

Categorical variables are summarized as frequencies with percentages, and were compared using the chi-square test or Fisher’s exact test. Continuous variables are shown as median (25^th^–75^th^ percentiles) and were compared using the Wilcoxon rank sum test. For outcome analysis, event-free survival was estimated by the Kaplan-Meier method and compared with the log-rank test. We used propensity score-matched analysis to balance intergroup differences. The propensity score, which represents the probability of use of IV morphine, was estimated without regard to outcome using multiple logistic regression analysis. A fully non-parsimonious model was developed that included all variables shown in Table 1. Those variables included age, sex, current smoking, diabetes mellitus, hypertension, dyslipidemia, body mass index, Killip class, systolic blood pressure, heart rate, symptom to balloon time, door to balloon time, LV ejection fraction, anterior infarction, initial TIMI flow 0 or 1, vessel disease, stenting, and use of glycoprotein IIb-IIIa inhibitor. Upon matching propensity scores, the pairs were created using the nearest neighbor method. The adequacy of the propensity score-matched analysis was evaluated by the overall balance achieved in terms of a less than 0.1 standardized mean difference. Since balance was achieved, the matched data set was analyzed using the paired t-test for continuous variables and the McNemar’s test of symmetry for categorical variables. All tests were two sided, and p-values less than 0.05 were considered statistically significant. Logistic regression models were used to determine the independent predictors of high MSI (>44% [median]). Variables with p value <0.2 in univariate analysis and IV morphine use were included in multivariate analysis. All statistical analyses were performed using R 3.2.5 (R foundation for Statistical Computing, Vienna, Austria).

**Table 1:** Baseline characteristics.

	Total population			Propensity score-matched population		
	Morphine (+) (n = 96)	Morphine (-) (n = 203)	SMD (%)	Morphine (+) (n = 90)	Morphine (-) (n = 90)	SMD (%)
Age (years)	58.5 (50.0–66.0)	60.0 (52.0–70.0)	-28.1	59.0 (50.0–66.8)	58.0 (50.2–65.0)	-0.4
Male	77 (80.2)	164 (80.8)	-1.5	73 (81.1)	72 (80.0)	2.9
Current smoking	53 (55.2)	87 (42.9)	24.7	50 (55.6)	47 (52.2)	6.7
Diabetes mellitus	18 (18.8)	50 (24.6)	-14.8	17 (18.9)	16 (17.8)	2.8
Hypertension	37 (38.5)	91 (44.8)	-13.0	37 (41.1)	36 (40.0)	2.3
Dyslipidemia	15 (15.6)	31 (15.3)	0.8	15 (16.7)	14 (15.6)	3.0
Body mass index (kg/m^2^)	24.2 (21.9–26.5)	24.6 (22.3–26.8)	-12.0	24.2 (22.0–26.6)	24.3 (22.2–26.4)	7.7
Killip class II to IV	9 (9.4)	21 (10.3)	-3.1	9 (10.0)	7 (7.8)	7.5
Systolic blood pressure (mmHg)	131 (113–154)	133 (116–154)	-4.4	131 (115–154)	132 (117–152)	-0.4
Heart rate (bpm)	75 (63–90)	77 (67–91)	-16.9	76 (63–88)	74 (66–88)	-3.4
Symptom to balloon time (min)	155 (100–330)	221 (123–404)	-74.4	154 (100–295)	173 (120–318)	2.9
Door to balloon time (min)	68 (51–82)	67 (50–80)	12.5	68 (51–80)	68 (50–80)	5.0
LV ejection fraction (%)*	50.9 (43.0–56.8)	54.0 (46.9–61.0)	-24.7	52.4 (44.5–56.9)	50.3 (46.0–57.0)	-1.7
Anterior infarction	53 (55.2)	97 (47.8)	14.9	48 (53.3)	44 (48.9)	8.9
Initial TIMI flow 0 or 1	85 (88.5)	156 (76.8)	36.7	80 (88.9)	78 (86.7)	6.9
1-vessel disease	54 (56.2)	111 (54.7)	3.0	50 (55.6)	49 (54.4)	2.4
2-vessel disease	32 (33.3)	61 (30.0)	7.0	30 (33.3)	30 (33.3)	0.0
3-vessel disease	10 (10.4)	31 (15.3)	-16.1	10 (11.1)	11 (12.2)	-3.6
Stenting	91 (94.8)	191 (94.1)	3.2	85 (94.4)	86 (95.6)	-4.9
Use of GPIIb-IIIa inhibitor	18 (18.8)	37 (18.2)	1.5	16 (17.8)	13 (14.4)	8.4

Values are reported as median (25^th^–75^th^ percentiles) or n (%). GP, glycoprotein; LV, left ventricular; SMD, standardized mean difference; TIMI, Thrombolysis in Myocardial Infarction.

*LV ejection fraction was not available in 2 patients (2.0%) in the morphine group.

## Results

Of the 299 patients included in the present study, 96 patients (32.1%) received IV morphine before primary PCI and the median dose of IV morphine was 5 mg (3–10 mg). Baseline characteristics are shown in Table 1. Patients treated with IV morphine had younger age (58.5 years versus 60.0 years, *P* = .049) and higher prevalence of current smoking (55.2% versus 42.9%, *P* = .048). In addition, patients who received IV morphine showed shorter symptom to balloon time (155 min versus 221 min, *P* = .02), a tendency for lower LV ejection fraction (50.9% versus 54.0%, *P* = .06), and higher prevalence of TIMI flow grade 0 or 1 prior to PCI (88.5% versus 76.8%, *P* = .02) compared with those who did not receive IV morphine. After propensity score matching, we created 90 matched pairs of patients. There were no significant differences in the baseline demographic and angiographic characteristics between both groups.

The results of angiographic and biochemical outcomes are displayed in Table 2. The presence of no-reflow showed no difference according to IV morphine administration. Post-procedural TIMI flow grade 3 and post-procedural MBG 2 or 3 was achieved similarly in both groups. Peak CK-MB level was higher in the morphine group than in the non-morphine group (204.0 ng/ml versus 148.2 ng/ml, *P* = .01). After propensity score-matched analysis, there was no significant difference in peak CK-MB level between the two groups.

**Table 2:** Angiographic and biochemical outcomes.

	Total population			Propensity score-matched population		
	Morphine (+) (n = 96)	Morphine (-) (n = 203)	*P* Value	Morphine (+) (n = 90)	Morphine (-) (n = 90)	*P* Value
No-reflow	6 (6.3)	12 (5.9)	>0.99	6 (6.7)	6 (6.7)	>0.99
Post-procedural TIMI flow 3	92 (95.8)	187 (92.1)	0.32	87 (96.7)	83 (92.2)	0.29
Post-procedural MBG 2 or 3	96 (100.0)	202 (99.5)	>0.99	90 (100.0)	90 (100.0)	-
Peak CK-MB (ng/ml)	204.0 (100.3–292.0)	148.2 (53.0–265.4)	0.01	198.7 (100.0–291.1)	182.5 (103.2–302.1)	0.78

Values are reported as median (25^th^–75^th^ percentiles) or n (%). CK-MB, creatine kinase-myocardial band fraction; MBG, myocardial blush grade; TIMI, Thrombolysis in Myocardial Infarction.

**Fig 3:**
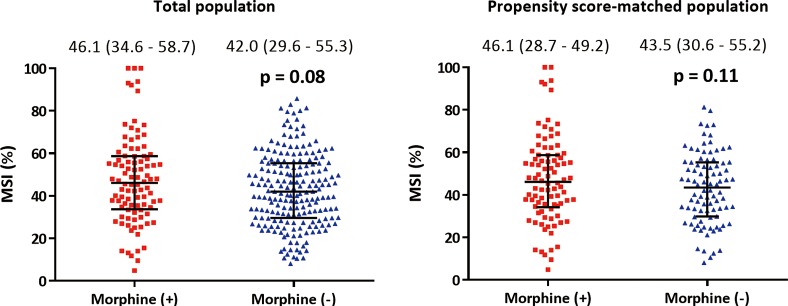
Myocardial salvage index in the total population and propensity score-matched population. Myocardial salvage index was greater in patients treated with morphine in crude analysis, but there was no significant difference between the two groups after propensity score-matched analysis.

There was no significant difference in the intervals from PCI to CMR between the two groups (3 [3–4] days in both groups, *P* = .88). Table 3 shows data analyzed from the cine, T2-weighted and contrast-enhanced CMR. The primary outcome, MSI, tended to be greater in the morphine group than the non-morphine group (46.1% versus 42.0%, *P* = .08) (Fig 3) as well as infarct size (22.2% versus 18.3%, *P* = .053). AAR (41.1% versus 33.4%, *P* < .001) and extent of MVO (3.7% versus 1.2%, *P* = .002) were significantly greater, and presence of hemorrhagic infarction (56.3% versus 39.4%, *P* = .01) or MVO (68.8% versus 56.2%, *P* = .04) was more frequent in patients treated with IV morphine than in patients without morphine treatment. However, after propensity score-matched analysis, both primary and secondary outcomes did not show any difference in both groups. MSI was similar (46.1% versus 43.5%, *P* = .11) as well as other CMR parameters including infarct size, AAR, presence of hemorrhagic infarction or MVO, and MVO volume in both groups.

**Table 3:** Cardiac magnetic resonance findings.

	Total population			Propensity score-matched population		
	Morphine (+) (n = 96)	Morphine (-) (n = 203)	*P* Value	Morphine (+) (n = 90)	Morphine (-) (n = 90)	*P* Value
LVEDV (ml)	145.1 (126.1–165.2)	143.0 (120.5–163.1)	0.56	145.1 (126.1–165.5)	146.9 (123.7–165.1)	0.95
LVESV (ml)	68.6 (53.5–84.7)	65.3 (49.9–81.2)	0.18	68.6 (53.3–84.5)	69.7 (55.1–86.3)	0.96
LV ejection fraction (%)	50.9 (44.1–57.9)	54.3 (46.9–60.9)	0.04	50.9 (44.1–58.4)	50.7 (44.8–57.3)	0.93
LV mass (g)	111.8 (99.9–125.7)	103.2 (91.2–122.2)	0.045	113.3 (100.2–127.8)	105.5 (90.9–123.2)	0.20
Infarct size (% of LV)	22.2 (12.9–29.6)	18.3 (12.4–25.8)	0.053	21.5 (12.8–28.1)	20.6 (14.4–29.7)	0.82
Area at risk (% of LV)	41.1 (28.8–52.3)	33.4 (22.6–42.3)	<0.001	40.6 (28.7–49.2)	38.6 (31.9–46.3)	0.48
Myocardial salvage index	46.1 (34.0–58.7)	42.0 (29.6–55.3)	0.08	46.1 (34.6–58.7)	43.5 (30.6–55.2)	0.11
Hemorrhagic infarction	54 (56.3)	80 (39.4)	0.01	51 (56.7)	44 (48.9)	0.38
MVO	66 (68.8)	114 (56.2)	0.04	62 (68.9)	57 (63.3)	0.53
MVO volume (% of LV)	3.7 (0.0–11.1)	1.2 (0.0–5.1)	0.002	3.7 (0.0–10.4)	1.9 (0.0–5.6)	0.16

Values are reported as median (25^th^–75^th^ percentiles) or n (%). LV, left ventricle; LVEDV, left ventricular end-diastolic volume; LVESV, left ventricular end-systolic volume; MVO, microvascular obstruction.

In multivariate logistic regression analysis, we identified that systolic blood pressure ≥140 mmHg was significantly associated with high MSI (Table 4).

**Table 4:** Predictors of high myocardial salvage index (>44%).

	Odds ratio	95% CI	*P* value
IV morphine use	1.19	0.72–1.96	0.49
Symptom to balloon time <300 mins	1.58	0.97–2.58	0.07
Body mass index ≥23 kg/m2	1.38	0.83–2.30	0.22
Killip class II to IV	0.63	0.29–1.38	0.25
Systolic blood pressure ≥140 mmHg	1.96	1.21–3.16	0.01

All variables included in multivariate analysis were listed in this table. CI, confidential interval; IV, intravenous.

The median follow-up duration was similar in both groups (20.6 months versus 28.4 months, *P* = .08 in total population; 24.2 months versus 25.9 months, *P* = .56 in propensity score-matched population). No significant differences in MACE-free survival were seen according to IV morphine use with or without propensity score-matched analysis (*P* = .18 for crude analysis and *P* = .26 for propensity score-matched analysis).

## Discussion

In the present study, we investigated whether IV morphine administration before PCI would affect myocardial salvage adversely in STEMI patients undergoing primary PCI. The major findings of our study were as follows: (1) patients in the morphine group showed greater MSI, larger infarct size, and higher prevalence of hemorrhagic infarction or MVO in the crude analysis, but (2) there was no significant difference in all these CMR parameters after propensity score-matched analysis. In short, IV morphine administration before PCI was not associated with adverse outcomes in myocardial salvage in our propensity score-matched analysis.

The use of IV morphine has been recommended as the treatment of choice for pain relief in patients presenting with STEMI [1,2] despite this being based only on expert opinion. On the other hand, recent studies have proposed adverse effects of IV morphine in their association with delayed activity of P2Y_12_ receptor inhibitors [3–6]. In the case of clopidogrel, for example, decreased plasma levels and diminished antiplatelet effects were reported in subjects who had received IV morphine [4]. This adverse effect of IV morphine was also observed in cases with other P2Y_12_ receptor inhibitors including prasugrel [3,6] and ticagrelor [5,6]. It has also been reported that decreased or delayed effects of antiplatelet agents could aggravate myocardial damage or limit myocardial salvage in AMI [16,17]. Because the importance of platelet inhibition cannot be overemphasized in such thrombotic circumstances, there has been a concern that IV morphine would result in reduction in myocardial salvage by impeding the action of P2Y_12_ receptor inhibitor in STEMI patients undergoing reperfusion therapy. Thus, we investigated the CMR images of STEMI patients undergoing primary PCI to determine the effects of IV morphine on myocardial injury.

Our propensity score-matched analysis showed that IV morphine was not associated with worse outcomes in terms of myocardial injury represented by MSI, infarct size, AAR, or MVO volume on CMR. In contrast to our results, a recent study reported adverse effects of IV morphine, including larger infarct size, higher extent of MVO, and lower MSI in the IV morphine group [18]. These contradictory results might be explained from the different statistical methods used to avoid selection bias in the observational study. Because patients received IV morphine in a non-randomized fashion, it is highly likely that patients with severe pain, compromised circulation or large infarct size were treated with IV morphine. In fact, the prevalence of TIMI flow grade 0 or 1 and peak CK-MB level was significantly higher in morphine group in our study and there was a trend toward higher prevalence of TIMI flow grade 0 or 1 in the morphine group of the other previous study (55.3% versus 44.4%, *P* = .09). While we performed propensity score-matched analysis to overcome these limitations, the previous study did not balance the differences in baseline variables between morphine and non-morphine groups.

Along with our result, real world data are somewhat conflicting with those pharmacokinetic/pharmacodynamic studies [18–21]. A recent cohort study reported that pre-hospital morphine use in STEMI patients was not associated with worse in-hospital complications and 1-year mortality [21]. It is likely that the findings of pharmacokinetic/pharmacodynamic studies cannot be simply extended to clinical outcomes. This kind of discrepancy has also been observed in other instances, such as with proton pump inhibitors and clopidogrel interactions [22]. Apart from its analgesic properties, morphine administration decreases heart rate, blood pressure, and venous return by reduced sympathetic activity. Based on these beneficial effects, morphine would reduce myocardial oxygen demand in critically ill patients [23]. Nevertheless, considering these conflicting results, a randomized controlled trial will be required to document the effects of IV morphine on patients with STEMI.

There were several limitations in this observational study. First, administration of IV morphine or glycoprotein IIb-IIIa inhibitors was left to the discretion of a clinician. To avoid selection bias and potential confounding effects, we performed propensity score-matched analysis. After propensity score matching, the 2 groups were well-balanced (standardized mean difference <10%), and we assumed that the effect of the covariate on the patients treated with or without morphine was similar. Second, we only used clopidogrel for dual antiplatelet therapy as an addition to aspirin. Clopidogrel is a widely used P2Y_12_ receptor inhibitor, but more potent antiplatelets such as ticagrelor or prasugrel are being increasingly recommended as alternatives [1,2]. We could not evaluate the effect of these agents in this study and future studies examining the different types of P2Y_12_ receptor inhibitors are required. Lastly, there was some variation in the interval between the index procedure and CMR, although there was no statistically significant difference in median time between the two groups.

In propensity score-matched analysis, IV morphine administration prior to primary PCI in STEMI patients did not increase the extent of myocardial damage or cause adverse impact on myocardial salvage assessed by CMR.

## Supporting Information

S1 Dataset Minimal relevant dataset of this study. (XLSX)
